# Gemcitabine reduces MDSCs, tregs and TGFβ-1 while restoring the teff/treg ratio in patients with pancreatic cancer

**DOI:** 10.1186/s12967-016-1037-z

**Published:** 2016-09-29

**Authors:** Emma Eriksson, Jessica Wenthe, Sandra Irenaeus, Angelica Loskog, Gustav Ullenhag

**Affiliations:** 1 Department of Immunology, Genetics and Pathology, Science for Life Laboratory, Rudbeck Laboratory C11 2nd floor, Uppsala University, Dag Hammarskjoldsvag 20, 751 85 Uppsala, Sweden; 2 Department of Oncology, Uppsala University Hospital, Uppsala, Sweden; 3 Lokon Pharma AB, Uppsala, Sweden

**Keywords:** Gemcitabine, Pancreatic cancer, Tregs, MDSCs, TGFβ

## Abstract

**Background:**

Cancer immunotherapy can be potentiated by conditioning regimens such as cyclophosphamide, which reduces the level of regulatory T cells (tregs). However, myeloid suppressive cells are still remaining. Accordingly to previous reports, gemcitabine improves immune status of cancer patients. In this study, the role of gemcitabine was further explored to map its immunological target cells and molecules in patients with pancreatic cancer.

**Methods:**

Patient blood was investigated by flow cytometry and cytokine arrays at different time points during gemcitabine treatment.

**Results:**

The patients had elevated myeloid-derived suppressor cells (MDSCs), and Tregs at diagnosis. Myeloid cells were in general decreased by gemcitabine. The granulocytic MDSCs were significantly reduced while monocytic MDSCs were not affected. In vitro, monocytes responding to IL-6 by STAT3 phosphorylation were prevented to respond in gemcitabine medium. However, gemcitabine could not prevent STAT3 phosphorylation in IL-6-treated tumor cell lines. TGFβ-1 was significantly reduced after only one treatment and continued to decrease. At the same time, the effector T cell:Treg ratio was increased and the effector T cells had full proliferative capacity during the gemcitabine cycle. However, after a resting period, the level of suppressor cells and TGFβ-1 had been restored showing the importance of continuous conditioning.

**Conclusions:**

Gemcitabine regulates the immune system in patients with pancreatic cancer including MDSCs, Tregs and molecules such as TGFβ-1 but does not hamper the ability of effector lymphocytes to expand to stimuli. Hence, it may be of high interest to use gemcitabine as a conditioning strategy together with immunotherapy.

**Electronic supplementary material:**

The online version of this article (doi:10.1186/s12967-016-1037-z) contains supplementary material, which is available to authorized users.

## Background

Pancreatic cancer is a devastating disease with an incidence rate that advances at a particularly high rate in industrialized countries. The commonly late diagnosis at an advanced stage and the resistance to conventional therapies make pancreatic cancer one of the most deadly cancers worldwide [1]. Pancreatic tumors have a characteristic immunosuppressive tumor microenvironment with dense stroma formation mediated by pancreatic stellate cells (PSCs). These cells get activated during inflammation by growth factors like transforming growth factor beta-1 (TGFβ-1), fibroblast growth factor and platelet-derived growth factor. Once activated, PSCs secrete collagen abundantly, which contributes to the dense and fibrous stroma formation. This process leads to the impaired vascularization and formation of the drug delivery barrier seen in pancreatic cancer [2]. Furthermore, PSCs can directly interact with immune cells as they secrete interleukin (IL)-6 and thus stimulate the recruitment of myeloid-derived suppressor cells (MDSCs) into the tumor microenvironment [3]. The immunosuppressive MDSCs represent a heterogeneous cell population of immature myeloid cells elevated in many cancers. MDSCs typically express the myeloid markers CD11b and CD33, but not the MHC class II molecule HLA-DR. They can be further subdivided into granulocytic or monocytic MDSCs based on CD14 expression [4]. MDSCs are expanded and activated in response to a variety of inflammatory factors that are secreted directly by tumor cells, PSCs and other adjacent immune cells. These factors include granulocyte–macrophage colony-stimulating factor (GM-CSF), TGFβ, interferon-gamma (IFN-γ) and a variety of interleukins (IL-1β, IL-6, IL-10) [5]. MDSCs are able to suppress the immune response mainly in two ways: They either directly suppress T cell responses or induce the expansion of regulatory T cells (Tregs). Both MDSCs and Tregs are present in early and late stage pancreatic tumors, whereas tumor-specific T cells are low in numbers and have an impaired activation [6]. Moreover, MDSCs and Tregs were also found to be positively and negatively correlated with tumor progression and overall survival, respectively [7, 8].

Immunotherapy is becoming a corner stone in cancer treatment with the introduction of checkpoint blockade antibodies [9]. However, significant responses in pancreatic cancer have thus far been absent [10, 11]. The severely immunosuppressive state combined with the dense profile of pancreatic lesions likely need to be combated to achieve an adequate anti-tumor immune response. In animal models, checkpoint blockade given together with other activating immunotherapy as well as gemcitabine ± paclitaxel have shown promising results [12, 13]. Gemcitabine is the standard of care for pancreatic cancer patients [14] and was shown to inhibit suppressor cells in these patients [15]. Therefore, combination of gemcitabine with immunotherapy in pancreatic cancer and potentially also in other indications may be of high interest. Hence, in this study we further explore the potential role of gemcitabine as a conditioning treatment to immunotherapy by evaluating the level of suppressor cells and their cytokines as well as the effector cell populations at different time points during gemcitabine treatment.

## Methods

### Patient samples

Blood samples were collected from patients diagnosed with pancreatic adenocarcinoma undergoing gemcitabine treatment at Uppsala University Hospital in accordance with ethical permit DNr: 2013/173. Gemcitabine is given at day 1, 8 and 15 in a 28-day cycle. Blood samples were collected just prior to gemcitabine infusion at day 1, 8, and 15 as well as after the resting phase (day 29) prior to the initiation of the next gemcitabine cycle. Blood taken in heparinized tubes was Ficoll-Paque gradient (GE Health Care, Little Chalfont, UK) separated and the peripheral mononuclear cells (PBMCs) were frozen at −80 °C. Plasma was collected from EDTA coated tubes and frozen at −80 °C. PBMCs from healthy controls (HCs) were collected from the blood bank at Uppsala University Hospital.

### Flow cytometry

Phenotypic expression of PBMCs from pancreatic cancer patients and HCs were evaluated with flow cytometry. In short, PBMCs were thawed and FcR blocking reagent (Miltenyi Biotec, Bergisch Gladbach, Germany) was added to block unspecific binding for 10 min. Cells were then incubated with specific antibodies and isotype control for 30 min at 4 °C before suspended in 0.5 % bovine serum albumin (BSA) (Sigma-Aldrich, St. Louis, USA) in 1× PBS. Cells stained for FOXP3 were fixed and permeabilized with FOXP3 fix/perm buffer set from Biolegend (San Diego, CA, USA) according to manufacturer’s protocol. Antibodies used: αCD11b PE/Cy7 (clone ICRF44), αCD14 APC/Cy7 (clone HCD14), αCD33 PE (clone WM53), αCD40 APC (clone HB14), αHLA DR PerCP (clone L243), αPD-L1 Brilliant Violet 421 (clone 29E.2A3), αCD3 FITC (clone UCHT-1), αCD16 PE/Cy7 (clone 3G8), αCD56 APC (clone HCD56), αCD4 PerCP (clone OKT4), αCD127 Brilliant Violet 421 (cloneA019D5), αFOXP3 Alexa Flour 649 (clone 206D), αCD107a PE (clone H4A3), mouse IgG1κ PE (clone MOPC-21), mouse IgG1κ APC (clone MOPC-21), mouse IgG1κ FITC (clone MOPC-21), mouse IgG2a PerCP (clone MOPC-173), mouse IgG2bκ Brilliant Violet 421 (clone MPC-11), mouse IgG1κ Alexa Flour 649 (clone MOPC-21), all purchased from Biolegend, and αCD86 FITC (clone 2331) purchased from BD Bioscience (San Jose, CA, USA). Cells were run in a BD FACS Canto II (BD Biosciences) and results were evaluated in Flow Jo (Tree Star, Ashland, OR, USA). For gating strategies see Additional file 1.

### STAT3 assay

Pancreatic cell lines MiaPaCa2 and Panc01, or donor-derived monocytes from two healthy donor buffy coats (Uppsala University Blood Central) were cultured ±IL-6 (10 ng/mL; Biolegend) and/or ±gemcitabine (20uM; Sigma-Aldrich, St. Louis, MO, USA) for 20 min and analyzed for STAT3 phosphorylation using Bio-plex Pro p-STAT 3 (Tyr705) kit from Bio-Rad (Hercules, CA, USA). Samples were run in triplicates. The experiment was repeated using a 4-h prestimulation with gemcitabine prior to IL-6 addition with similar results.

### Proliferation assay

The proliferation capacity of the PBMCs from pancreatic patients and HCs was evaluated with Alarmar Blue assay (Life Technologies™ Carlsbad, CA, USA). In short, PBMCs were thawed and adjusted to a concentration of 0.5 × 10^6^/mL in Roswell Park Memorial Institute 1640 medium supplement with 10 % fetal bovine serum and 1 % Penicillin Streptomycin from Life Technologies™. 100,000 cells together with 100 IU/mL of IL-2 (Proleukein, Novartis, Basel, Switzerland) were added into 96-well plates pre-coated with 1 µg/mL of OKT-3 (Biolegend). Measurements were taken at 0, 24, 48, 72 and 96 h at 570 nm and 595 nm as background.

### Cytokine analysis

Plasma from the pancreatic adenocarcinoma patients were evaluated by ELISA for expression of TGF-β1 (Diaclone, Besançon cedex, France), Arginase-1 (Hycult^®^ biotech, Plymouth Meeting, PA, USA), and Myeloperoxidase (Biolegend). Mesco Scale Discovery V-plex (Rockville, MD, USA) was used for detection of IL-1β, IL-6, IL-8, IL-10, and TNFα.

### Statistical analysis

Graphpad Prism (Graphpad Software Inc. La Jolla, CA, USA) was used for determining statistical significance. Differences between HCs and patients were assessed by unpaired *t* test with Welch’s correction. Wilcoxon matched-pairs signed-rank test was performed to evaluate differences between the patient samples of the distinct days. STAT3 analyses were done using one-way ANOVA for multi-comparison testing.

## Results

### Patient characteristics

**Fig. 1:**
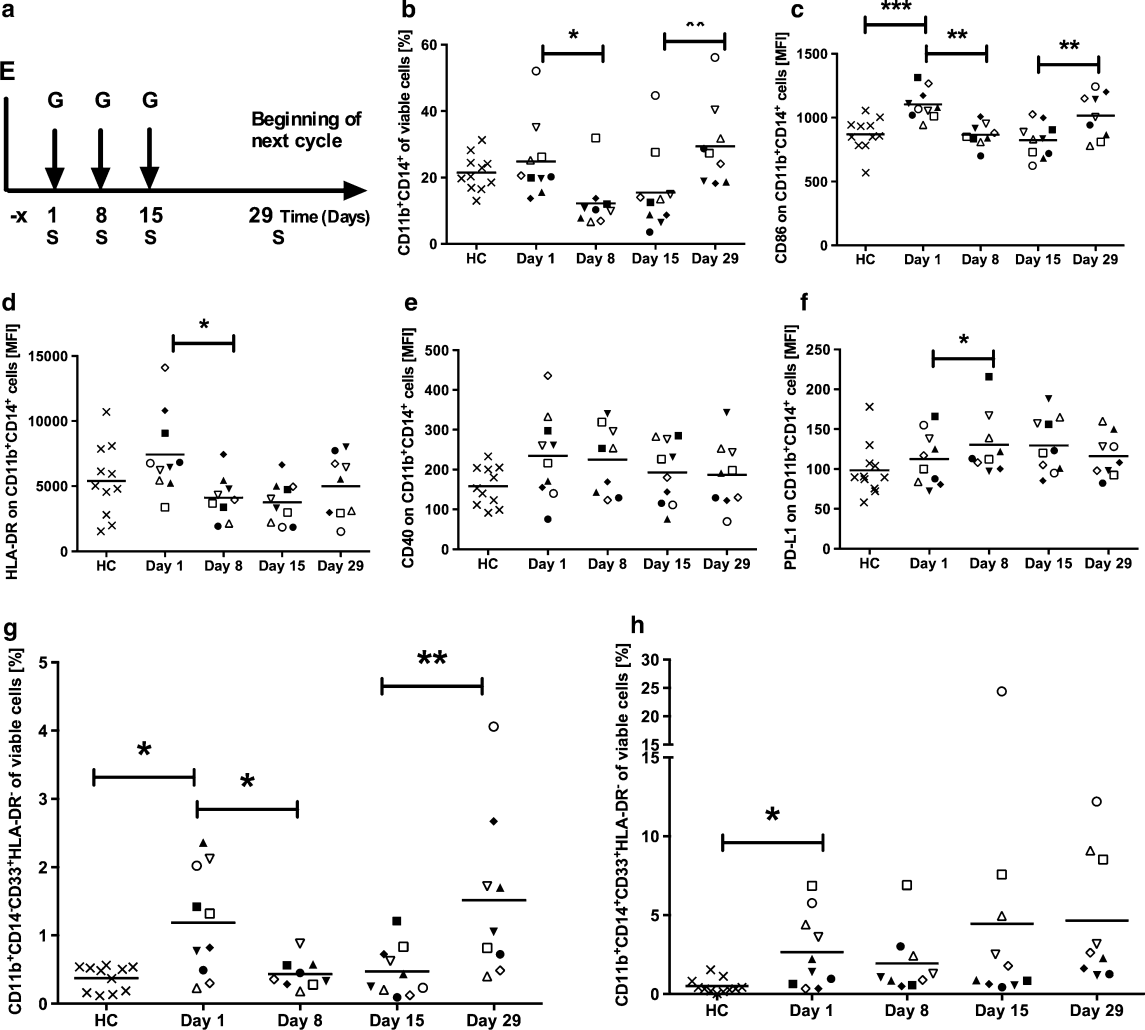
Study outline and the effect of gemcitabine on myeloid cells. **a** Ten pancreatic cancer patients were enrolled (*E*) and sampled during the first cycle of gemcitabine treatment (*G*), where gemcitabine was given once weekly for three weeks, followed by a resting period. Blood samples (*S*) were collected at day 1, 8, 15 and 29 always before gemcitabine was given that day. Patient and healthy control (HC) samples were stained for CD14^+^CD11b^+^ monocytes (**b**) and CD86 (**c**), HLA-DR (**d**), CD40 (**e**) and PD-L1 expression (**f**). Samples were also stained for granulocytic (**g**) and monocytic MDSCs (**h**), defined as CD11b^+^CD14^−^ CD33^+^ HLA-DR^−^ and CD11b^+^ CD14^+^ CD33^+^ HLA-DR^-^, respectively. Samples were analyzed by flow cytometry. Statistical differences between HCs and patients or between sample days were assessed by unpaired t-test with Welch’s correction or Wilcoxon matched-pairs signed-rank test, respectively (*P < 0.05, **P < 0.01, ***P < 0.001)

In this study, ten patients diagnosed with pancreatic adenocarcinoma were enrolled to receive gemcitabine treatment as their standard of care and were investigated for their immune profile before and at different time points post treatment initiation. The patient group consisted of six men and four women, with a mean age at study entry of 70.3 ± 6 years and 71.3 ± 6.1 years, respectively (Additional file 2). Three patients had their tumor removed by surgery and then received adjuvant gemcitabine treatment. Seven patients received gemcitabine as palliative care without prior surgery, of which five patients were treated for locally advanced disease and two patients for metastasized disease. Additional file 2: Table S1 displays the clinical characteristics of each patient. The patients were treated with gemcitabine (1000 mg/m^2^) once weekly for 3 weeks (day 1, 8, 15) followed by a resting phase for 2 weeks after the third treatment (Fig. 1a).

### Gemcitabine reduces myeloid cells including granulocytic myeloid-derived suppressor cells

Gemcitabine is known to affect myeloid cells. In our study, the level of CD11b^+^CD14^+^ monocytes was similar between the patients and healthy controls prior to treatment (day 1). After the first gemcitabine administration, the monocytes were significantly reduced but were restored after the resting period (Fig. 1b). CD86 was significantly higher expressed on patients’ monocytes at day 1 compared to controls. Nevertheless, the expression of CD86 (Fig. 1c) was decreased after treatment with gemcitabine but increased after the resting week. A similar trend was observed for the expression of the MHC class II molecule HLA-DR (Fig. 1d). In contrast, patient monocytes exhibited no significant change of CD40 expression (Fig. 1e), while expression of PD-L1 (Fig. 1f) was significantly increased after the first gemcitabine administration.

MDSCs are generally classified as either belonging to the granulocytic or monocytic lineage. In humans, MDSCs are mostly referred to as the granulocytic subset, meaning that they do not express CD14 but express CD11b and CD33 and lack the expression of the MHC class II molecule HLA-DR [5]. Thus, we defined the granulocytic subset as CD11b^+^CD14^−^CD33^+^HLA-DR^−^ and also investigated a monocytic subset (CD11b^+^CD14^+^CD33^+^HLA-DR^−^). Both the granulocytic (Fig. 1g) and monocytic (Fig. 1h) MDSCs were significantly elevated in the patient population compared to healthy controls before treatment. After the first treatment, the granulocytic MDSCs were significantly decreased to a level similar to controls. However, after the resting period, the granulocytic MDSCs were increased again. Despite the reduction of granulocytic MDSCs, gemcitabine did not affect monocytic MDSCs. The patients with the shortest survival had significantly higher levels of monocytic MDSCs compared to patients who survived more than 1 year.

### The effector T cell:T regulatory cell ratio is shifted by gemcitabine

**Fig. 2:**
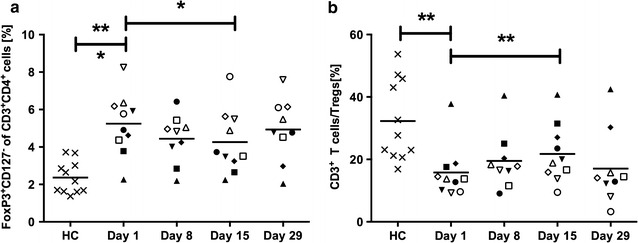
The effect of gemcitabine on regulatory T cells (Tregs). Ten pancreatic cancer patients were sampled during the first cycle of gemcitabine treatment, where gemcitabine was given once weekly for 3 weeks, followed by a resting period. Blood samples were collected at day 1, 8, 15 and 29 always before gemcitabine was given that day. Patient and healthy control (HC) samples were stained for tregs, which were defined as CD3^+^CD4^+^CD127^−^FoxP3^+^. Samples were analyzed by flow cytometry. Statistical differences between HCs and patients or between sample days were assessed by unpaired t-test with Welch’s correction or Wilcoxon matched-pairs signed-rank test, respectively (*P < 0.05, **P < 0.01, ***P < 0.001)

Tregs, defined as CD3^+^CD4^+^CD127^−^FoxP3^+^ cells [16], were significantly elevated in patients before treatment compared to healthy individuals. Gemcitabine treatment reduced the mean Tregs number modestly but significantly, since almost all patients showed a decrease in Tregs from baseline. Nevertheless, the Treg level in patients was still elevated in comparison to the healthy controls at all time points (Fig. 2a). Lymphocytes are generally not affected by gemcitabine. However, the ratio between effector T cells and Tregs was elevated at day 15, indicating a reduction of Tregs and/or an increase of effector T cells subsequent to gemcitabine therapy (Fig. 2b).

### TGFβ-1 is reduced by continuous gemcitabine administration

**Fig. 3:**
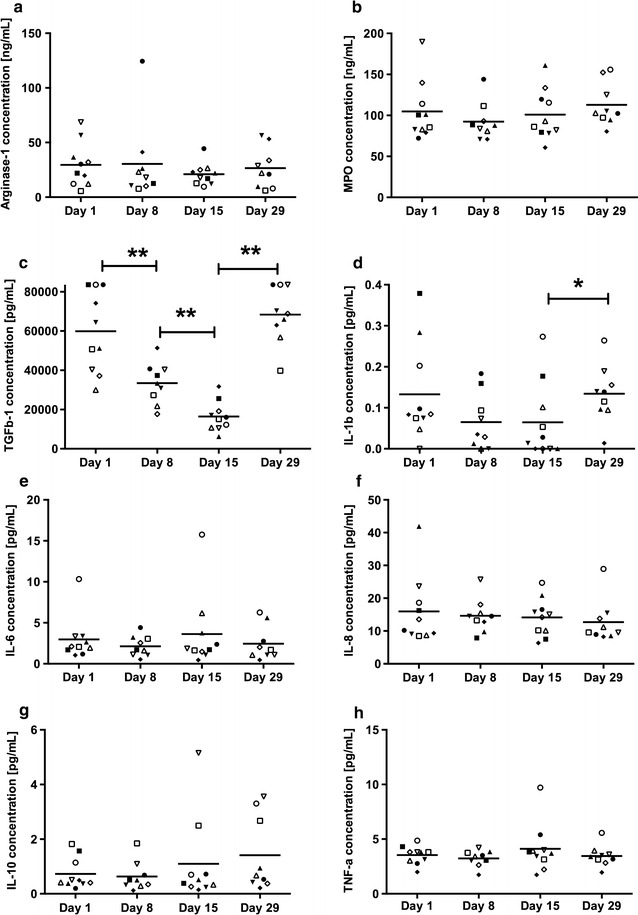
The effect of gemcitabine on immunomodulatory plasma proteins. Ten pancreatic cancer patients were sampled during the first cycle of gemcitabine treatment, where gemcitabine was given once weekly for 3 weeks, followed by a resting period. Blood samples were collected at day 1, 8, 15 and 29 always before gemcitabine was given that day. Plasma levels of Arginase-1, MPO and TGFβ-1 were evaluated by ELISA (**a**–**c**) and MDS multiplex analysis was performed for determining plasma levels of IL-1β, IL-6, IL-8, IL-10 and TNFα (**d**–**h**). Concentrations are displayed in ng/mL or pg/mL and statistical differences were assessed by Wilcoxon matched-pairs signed-rank test (*P < 0.05, ** P < 0.01)

To further evaluate the effect of gemcitabine on the patients’ immunosuppressive status, plasma samples were assessed for inflammatory molecules (Fig. 3). The mean values of arginase-1, myeloperoxidase (MPO), IL-6, IL-8, IL-10 and TNFα did not change considerably throughout the first cycle of gemcitabine treatment even if several individual patients had decreased Arginase-1 and MPO post gemcitabine initiation. TGFβ-1 was significantly reduced by gemcitabine in all patients. The level of TGFβ-1 decreased already after the first administration and was even further reduced at day 15. Despite this immense reduction, TGFβ-1 was restored to the initial level after the resting period (day 29) (Fig. 3c). A significant increase after the resting period was observed in IL-1β. However, overall IL-1β was very low (Fig. 3d).

### The effect of gemcitabine on STAT3

**Fig. 4:**
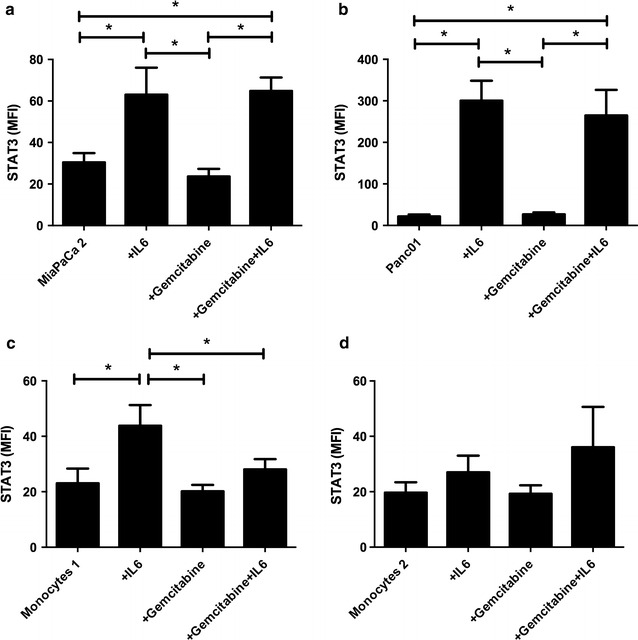
STAT3 phosphorylation post IL-6 and gemcitabine treatment. Pancreatic cell lines MiaPaCa2 (**a**) and Panc01 (**b**) or healthy donor derived monocytes from donor 1 (**c**) or donor 2 (**d**) were cultured ±IL-6 (10 ng/ml) and/or ±gemcitabine (20 μM) and analyzed for STAT3 phosphorylation using Bio-plex Pro p-STAT3 (Tyr705) kit. Samples were run in triplicates. Statistical differences were calculated using one-way ANOVA multicomparison test at a 95 % confidence interval. Significant differences are labeled with an *asterisk*

STAT3 is involved in the regulation of myeloid cells toward a more suppressive phenotype and since the myeloid population was affected by gemcitabine the level of phosphorylated STAT3 was evaluated in tumor cells and monocytes in vitro in response to gemcitabine. To induce STAT3 phosphorylation tumor cells and monocytes were stimulated by IL-6, cultured with or without gemcitabine and then analyzed by using Bio-plex Pro p-STAT3 (Tyr705) kit (Fig. 4). IL6 induced STAT3 phosphorylation in both MiaPaCa2 and Panc01 pancreatic cancer cell lines, but only in one of the two healthy donor-derived monocytes. In both tumor cell lines, the addition of gemcitabine to the IL-6-stimulated cells did not decrease STAT3 phosphorylation. However, in the monocyte culture that initially responded to IL-6, gemcitabine prevented STAT3 phosphorylation while the other donor showed no difference to gemcitabine.

### Gemcitabine does not affect the T cell proliferative capacity

**Fig. 5:**
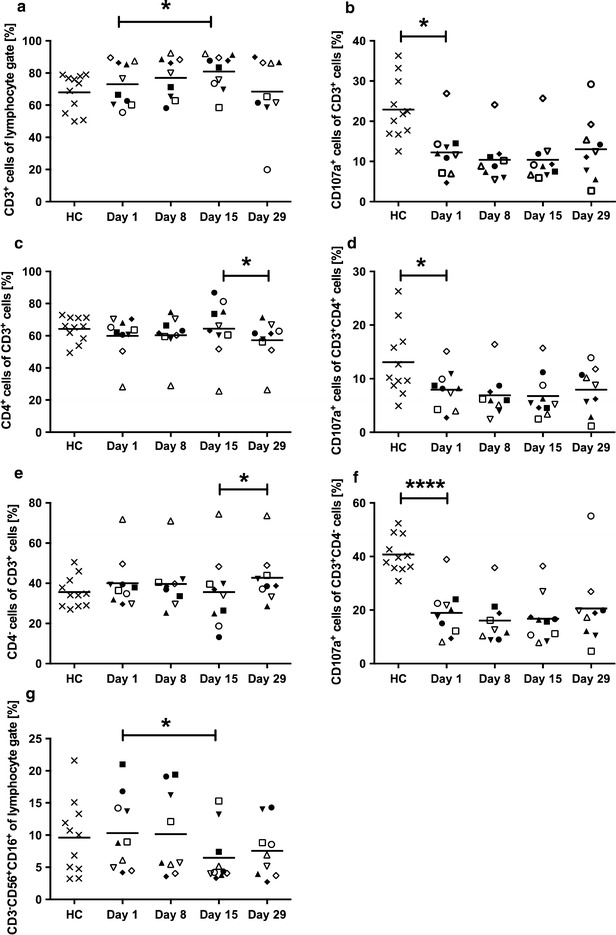
The effect of gemcitabine on effector lymphocytes. Ten pancreatic cancer patients were sampled during the first cycle of gemcitabine treatment, where gemcitabine was given once weekly for 3 weeks, followed by a resting period. Blood samples were collected at day 1, 8, 15 and 29 always before gemcitabine was given that day. Patient and healthy control (HC) samples were stained for CD3^+^ lymphocytes, CD4^+^ and CD4^−^ T cells (CD8^+^) and NK cells (CD56^+^CD16^+^). T cell activation was assessed by CD107a expression and the samples were analyzed by flow cytometry. Statistical differences between HCs and patients or between sample days were assessed by unpaired t-test with Welch’s correction or Wilcoxon matched-pairs signed-rank test, respectively (*P < 0.05, ****P < 0.0001)

In order to exclude that gemcitabine treatment depletes T and NK cells, which are crucial for anti-tumor immune responses, their presence was investigated before (day 1) and at different time points post gemcitabine treatment initiation (day 8, 15, 29). T cells were gated as CD3^+^ lymphocytes and further divided into T helper cells (CD3^+^CD4^+^) and cytotoxic T cells (CD3^+^CD4^−^). At day 1, both T cells and NK cells were at the same level as in healthy controls (Fig. 5a, c, e, g). Throughout gemcitabine treatment, CD3^+^ lymphocytes were significantly increased from day 1 to day 15. The level of T helper cells declined after the resting period while the cytotoxic T cells were increased (Fig. 5c, e). Nevertheless, both T helper cells and cytotoxic T cells had a decreased base level of CD107a, which is a marker of cytolytic activity, compared to T cells from healthy controls. However, CD107a expression remained stable during gemcitabine treatments (Fig. 5b, d, f). There was a tendency of increased CD107a expression in the cytotoxic T cell population at day 29 but it did not reach significance. NK cells were gated as CD3^−^CD56^+^CD16^+^ lymphocytes. The level of NK cells in the patients was similar to healthy controls (Fig. 5g). NK cells seemed more sensitive to gemcitabine than T cells, and the NK cell level was reduced in most patients from day 1 to day 15.

**Fig. 6:**
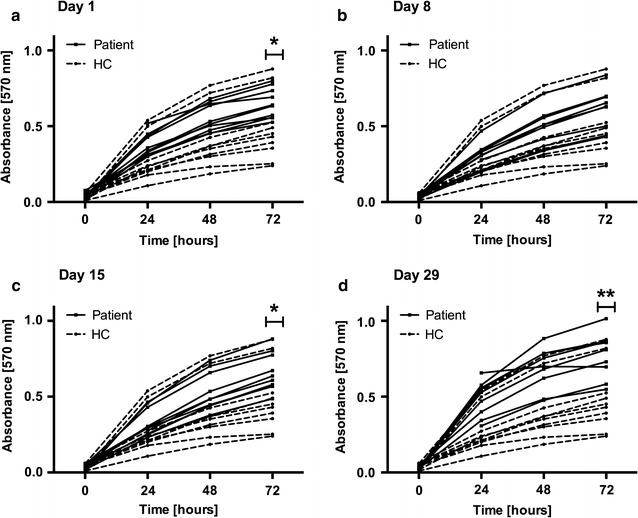
Proliferative capacity of T cells post gemcitabine. Ten pancreatic cancer patients were sampled during the first cycle of gemcitabine treatment, where gemcitabine was given once weekly for 3 weeks, followed by a resting period. Blood samples were collected at day 1, 8, 15 and 29 always before gemcitabine was given that day. The capacity of T cells to proliferate in response to CD3 antibody and IL-2 stimulation was assessed by Alarmar Blue assay. Measurements were taken at 0, 24, 48, and 72 h at 570 and 595 nm. *Grey lines* Healthy controls (HC), *Black lines* patients. Statistical differences between HCs and patients were assessed by unpaired t-test with Welch’s correction (*P < 0.05, **P < 0.01)

Finally, the capacity of the T cells to proliferate was evaluated to ensure that the patients’ T cells are able to respond to stimuli during gemcitabine treatment. PBMCs from both patients and healthy controls were stimulated with anti-CD3 (OKT-3) and IL-2 and their proliferative response was evaluated by Alamar Blue proliferation kit over time. The patients’ T cells responded as good as T cells from healthy controls at any time point after gemcitabine initiation. In fact, in comparison to the controls, the patients’ T cells proliferated slightly better and this was most evident in patient samples from day 29 (Fig. 6).

## Discussion

Despite other advances in cancer research, the poor prognosis for pancreatic cancer patients has not changed during the last few decades and life expectancy remains low [17]. Recently, immunotherapy has been in the limelight of cancer research, where impressive effects have been seen using immune checkpoint antibody blockade treatment in various malignancies and the treatment has been established in melanoma patients [18]. Such antibodies improve T cell function by blocking CTLA-4 or PD-1/PD-L1 signaling which releases the anergic T cells to become active participants in the anti-tumor immune response [19]. In a phase 2 trial by Royal et al., the effect of the monoclonal anti-CTLA-4 antibody ipilimumab was studied in patients with advanced pancreatic cancer. Only one patient out of 27 showed a delayed response to ipilimumab, indicating that this strategy is not of significant value for this indication [11]. However, animal data suggests that it may be possible to circumvent checkpoint blockade resistance by adding immunostimulatory agents. For example, checkpoint blockade in combination with agonistic CD40 antibodies plus gemcitabine and nab-paclitaxel showed promising data in mice [13]. In the experimental models, chemotherapy improved the results on tumor growth. Studies are ongoing to explore if immunotherapy successfully can be given simultaneously to the standard of care gemcitabine in patients with pancreatic cancer (http://www.clinicaltrials.gov). The doses, the route of administration and effect over time is not comparable in mice and man.

In this study, we investigated the immunomodulatory properties of gemcitabine chemotherapy in ten pancreatic cancer patients. Even if the cohort was small, the results were consistent showing the same trend in most patients and, hence, small variations. Myelosuppression is a well-known side effect of chemotherapies [20]. Thus, also we observed that myeloid cells including MDSCs were reduced by gemcitabine treatment. Chikamatsu et al. proposed that CD86 and PD-L1 are implicated in mediating the suppressive effects of MDSCs [21]. We found that gemcitabine treatment could reduce CD86, but not PD-L1 expression, which instead increased subsequently to the first gemcitabine administration. Hence, it might be of interest to combine gemcitabine chemotherapy with PD-1/PD-L1 blockade. Gemcitabine treatment also significantly reduced CD11b^+^CD14^−^CD33^+^HLA-DR^−^ granulocytic MDSCs, although this reduction was reversed after the resting phase where no gemcitabine was given. This demonstrates the need to administer gemcitabine continuously to achieve a durable effect on the levels of MDSCs. A combination of gemcitabine and capecitabine was previously shown to reduce Lin-DR^−^CD11b^+^ MDSCs in 42 % of the studied pancreatic cancer patients, but this reduction did not reach significance [22]. In our study, monocytic MDSCs were the most prevalent in the peripheral blood, but this subset was not significantly reduced by gemcitabine. Also, these cells were present at higher levels in patients who survived less than 1 year. Opposed to this, Khaled et al. only found granulocytic MDSCs (Lin^−^HLA-DR^−^CD33^+^CD11b^+^CD15^+^), but not monocytic MDSCs (Lin^−^HLA-DR^−^CD14^+^) to be elevated in pancreatic cancer patients [23]. These contradictory results might be explained by the heterogeneity of MDSC markers used to define the different subsets.

Despite the reduction of granulocytic MDSCs subsequent to gemcitabine treatment, we found no significant change of the MDSC effector molecules arginase-1 and MPO. However, values in several individual patients decreased even if that did not significantly alter the whole cohort. A decrease of these markers along with a decrease of MDSCs has been achieved with other anti-cancer drugs, for example with tyrosine kinase inhibitors in myeloid leukemia patients [24]. Nevertheless, in individual patients we did see a decrease of these inhibitors. A study by Khaled et al. showed that only granulocytic MDSCs expressed arginase-1 [23] and it has been proposed that monocytic MDSCs exert their suppressive function through inducible nitric oxide synthase [5]. In contrast, Filipazzi et al. demonstrated that monocytic CD14^+^HLA-DR^−/lo^ cells mediated their suppressive activity via TGFβ release [25]. In the current study, we found a profound effect of gemcitabine treatment on TGFβ-1 plasma level. TGFβ-1 was reduced subsequent to each administration, but was increased again after the resting period. In a study in pancreatic cancer patients by Vizio et al., TGFβ-1 was not significantly reduced by gemcitabine. However, these patients had overall much lower levels of TGFβ-1 [15]. This and different sampling time points might explain the contrasting results. In patients with non-small cell lung cancer, treatment with gemcitabine plus cisplatin significantly reduced serum TGF-β1 levels in patients who had a complete or partial response to chemotherapy [26].

The strong inhibitory effect of gemcitabine on TGFβ-1 may be explained by regulation by STAT3. STAT3 is a transcription factor that plays a crucial role in many essential processes of the cell, including survival and proliferation [27]. Moreover, STAT3 signaling is particularly important in the expansion and function of MDSCs [5], in the conversion of naïve CD4^+^ T cells to Tregs [28] and also in the induction of TGFβ-1 [29]. In a study by Tang et al., gemcitabine was found to inhibit several genes that are regulated by STAT3, which suggests that gemcitabine, might indeed target STAT3 and its downstream signaling [30]. In addition, a connection between gemcitabine and STAT3 is plausible as inhibition of STAT3 was observed to enhance the response to gemcitabine in a pancreatic cancer animal model [31]. Our results indicate that gemcitabine may prevent STAT3 phosphorylation in myeloid cells while we did not see any significant difference in STAT3 phosphorylation upon gemcitabine treatment in pancreatic tumor cell lines. Hence, gemcitabine in clinically relevant levels [32] does not likely target STAT3 directly but may affect STAT3 phosphorylation by indirect mechanisms in myeloid cells. Due to the different responses noted in different donors, such events may also be individual and highly time point dependent.

Gemcitabine is not in general known to affect the lymphocyte population. Nevertheless, we evaluated the level of Tregs and effector T cells in our patients. Tregs were elevated in our cohort at pretreatment and this has also been noted in other studies [33, 34]. Furthermore, Treg depletion by gemcitabine has previously been described both in tumor-bearing mice and in cancer patients [15, 35, 36]. In our cohort of pancreatic cancer patients, gemcitabine reduced Treg levels modestly after two treatments. Nevertheless, gemcitabine did not hamper the function of effector T cells in our study. Rather, the effector to Treg ratio was increased and the proliferative capacity of effector T cells seemed higher in the patients compared to healthy controls, especially after completing the cycle of three gemcitabine doses. Correspondingly, Plate et al. noted an increase in CD4^+^ T cells and an enhanced T cell function during gemcitabine therapy in pancreatic cancer patients [37]. In another study, in which pancreatic cancer patients were treated with gemcitabine plus cisplatin, T cell function was not impaired [38]. The effect of gemcitabine on the lymphocytes is likely due to the reduction of immunosuppressive cells and TGFβ-1 rather than a direct effect on these cells.

## Conclusions

In conclusion, gemcitabine decreases immunosuppressive immune cell populations and reduces TGFβ-1 considerably while sparing the function of effector T cells. Hence, conditioning treatment with gemcitabine alongside of immunotherapy may be effective in pancreatic cancer patients for whom gemcitabine is golden standard, but possibly also in patients with other malignancies.

## Additional files

10.1186/s12967-016-1037-z Gating strategies for flow cytometry.
10.1186/s12967-016-1037-z Patient characteristics.

## Abbreviations

PSCs: pancreatic stellate cells
TGFβ-1: transforming growth factor beta-1
IL: interleukin
IFN-γ: interferon-γ
MDSCs: myeloid-derived suppressor cells
Tregs: regulatory T cells
PBMC: speripheral mononuclear cells
HC: healthy controls
BSA: bovine serum albumin
MPO: myeloperoxidase
