# Supplementary material for: Gemcitabine reduces MDSCs, tregs and TGFβ-1 while restoring the teff/treg ratio in patients with pancreatic cancer
Source: J Transl Med. 2016 Sep 29;14:282. doi: 10.1186/s12967-016-1037-z (PMC5041438; doi:10.1186/s12967-016-1037-z)
Supplement: Supplementary file 1 — 10.1186/s12967-016-1037-z Gating strategies for flow cytometry. [file 12967_2016_1037_MOESM1_ESM.pdf]

Supplementary Information 1

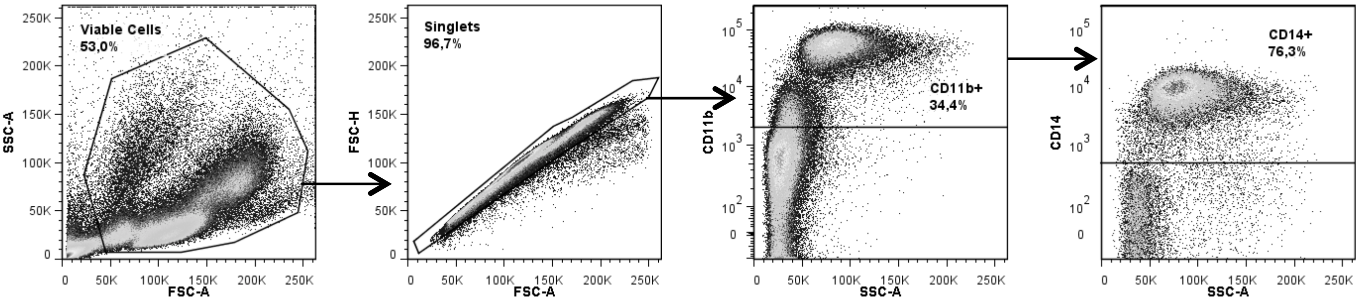

Gating strategy for monocytes. First, viable cells were gated based on forward (FSC) and sideward scatter (SSC) and singlets were gated from FSC area vs. FSC height. Subsequently, the singlets were gated for CD11b+ cells and then for CD14+ cells.

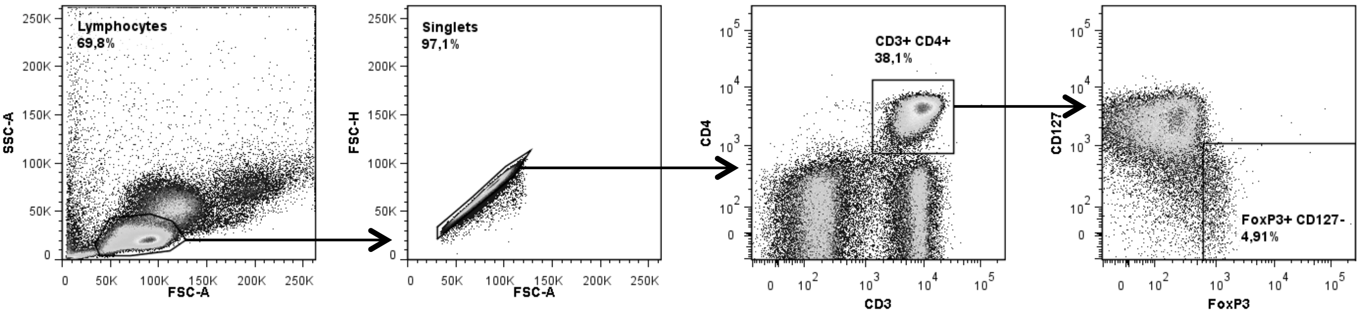

Strategy for gating regulatory T cells (Tregs). First, lymphocytes were gated based on forward (FSC) and sideward scatter (SSC) and singlets were gated from FSC area vs. FSC height. The single cell population was then gated for $CD3^{+}$ and $CD4^{+}$ cells. Lastly, these cells were gated for $FoxP3^{+}$ and $CD127^{-}$ based on cells stained with isotype control antibodies.

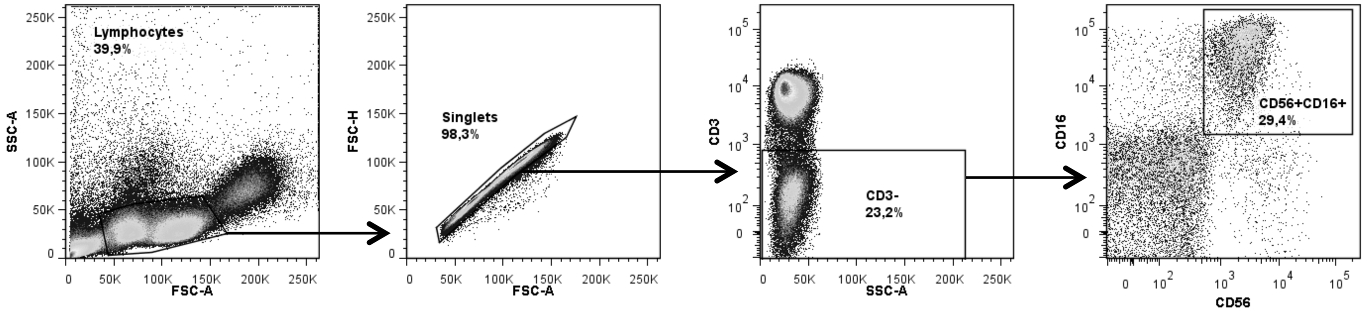

Gating strategy for NK cells. First, lymphocytes were gated based on forward (FSC) and sideward scatter (SSC) and singlets were gated from FSC area vs. FSC height. The single cell population was then gated for $CD3^{-}$ cells. Lastly, these cells were gated for expression of CD56 and CD16.

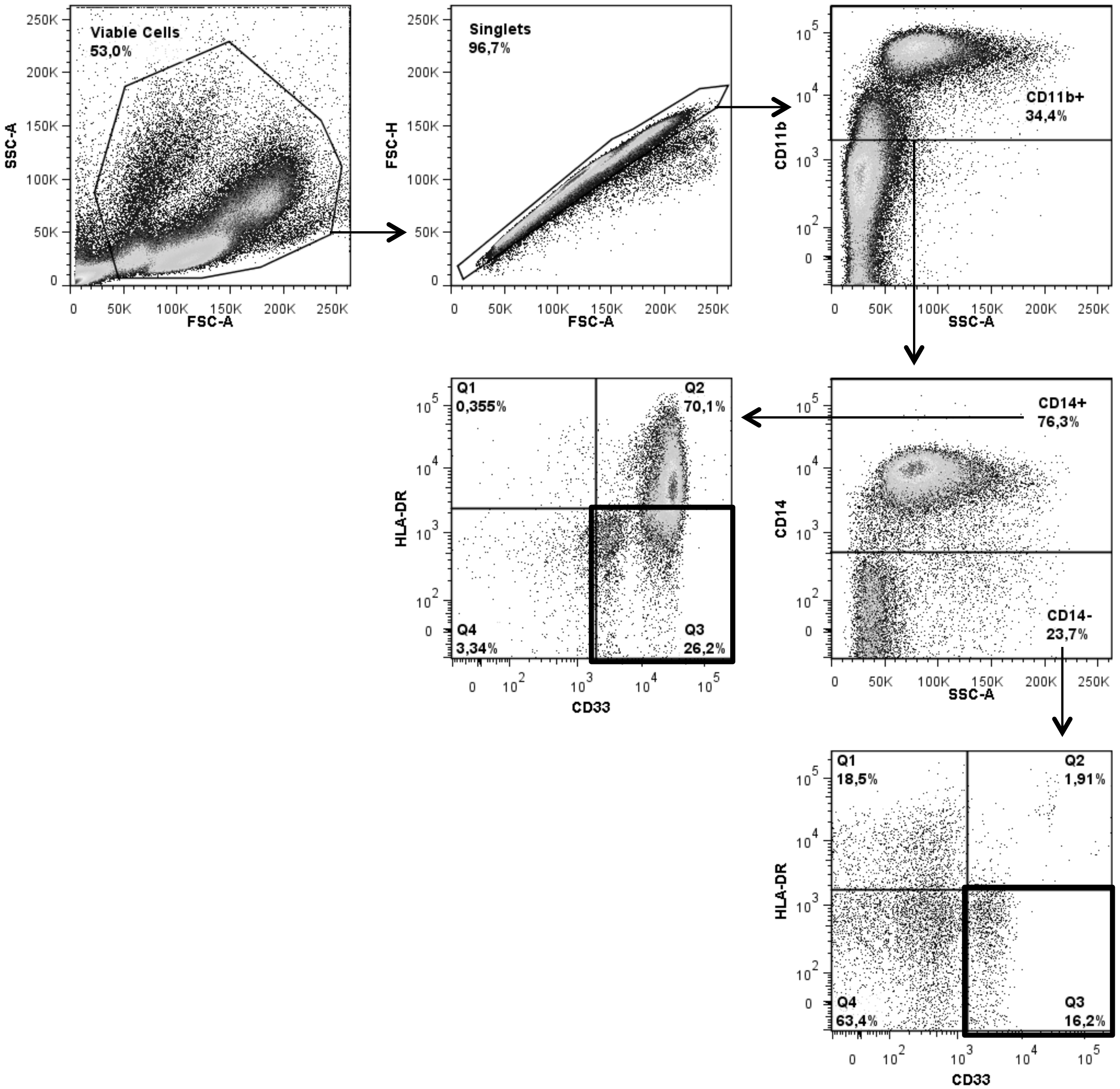

Strategy for gating myeloid-derived suppressor cells (MDSCs). First, viable cells were gated based on forward (FSC) and sideward scatter (SSC) and singlets were gated from FSC area vs. FSC height. Then $CD11b^{+}$ cells were gated from the single cells gate and divided into $CD14^{-}$ and $CD14^{+}$ populations. Finally, the resulting cell populations were gated for $CD33^{+}$ and $HLA\text{-}DR^{-}$ cells. The last gates were set in accordance with cells stained with isotype control antibodies.

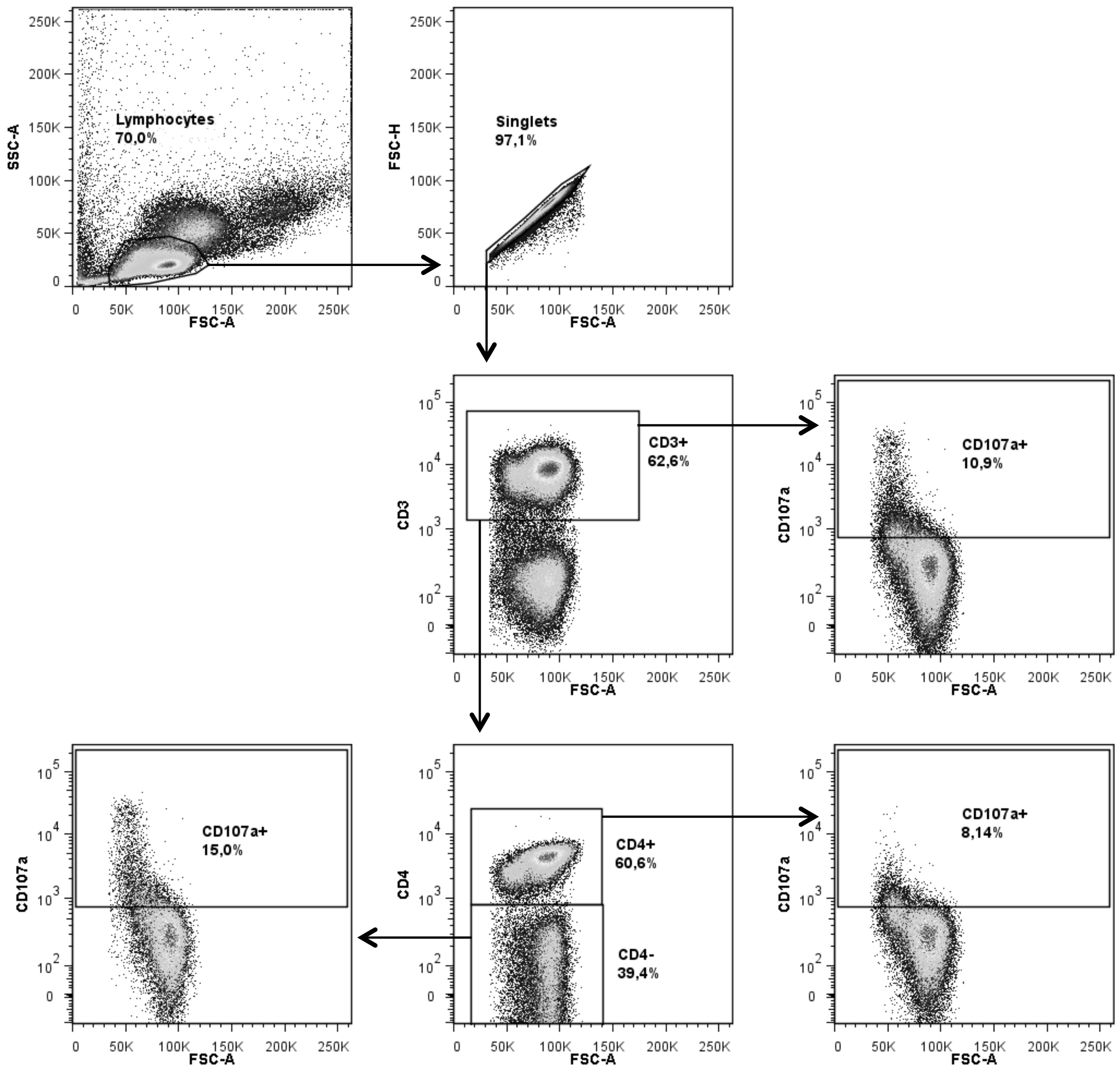

Strategy for gating T cells. First, lymphocytes were gated based on forward (FSC) and sideward scatter (SSC) and singlets were gated from FSC area vs. FSC height. The single cell population was then gated for $CD3^{+}$ cells and the expression of CD4. Lastly, these cell populations were gated for $CD107a^{+}$ cells.
