# Supplementary material for: Gemcitabine reduces MDSCs, tregs and TGFβ-1 while restoring the teff/treg ratio in patients with pancreatic cancer
Source: J Transl Med. 2016 Sep 29;14:282. doi: 10.1186/s12967-016-1037-z (PMC5041438; doi:10.1186/s12967-016-1037-z)
Supplement: Supplementary file 2 — 10.1186/s12967-016-1037-z Patient characteristics. [file 12967_2016_1037_MOESM2_ESM.pdf]

Supplementary Information 2: Patient characteristics						
**Patient**	**Sex**	**Age at inclusion**	**Stage at inclusion**	**Treatment**	**WHO* at inclusion**	**OS# (weeks)**
Patient 1	Male	66	Unresectable (Metastatic disease)	Palliative	1	48
Patient 2	Male	80	Resectable	Adjuvant	0	101
Patient 3	Female	76	Unresectable (Locally advanced)	Palliative	0 to 1	9
Patient 4	Male	65	Resectable	Adjuvant	0	61
Patient 5	Female	65	Resectable	Adjuvant	0	58
Patient 6	Male	66	Unresectable (Locally advanced)	Palliative	2 to 3	14
Patient 7	Female	77	Unresectable (Locally advanced)	Palliative	1	17
Patient 8	Male	75	Unresectable (Locally advanced)	Palliative	1	45
Patient 9	Female	67	Unresectable (Metastatic disease)	Palliative	1 to 2	64+
Patient 10	Male	70	Unresectable (Locally advanced)	Palliative	1	62+

*World Health Organization performance status score #Overall survival
